# Pullout Behavior of Recycled Waste Fishing Net Fibers Embedded in Cement Mortar

**DOI:** 10.3390/ma13184195

**Published:** 2020-09-21

**Authors:** Jun Kil Park, Min Ook Kim, Dong Joo Kim

**Affiliations:** 1 Coastal Development and Ocean Energy Research Center, Korea Institute of Ocean Science and Technology, Busan 49112, Korea; jkpark@kiost.ac.kr; 2 Department of Civil and Environmental Engineering, Sejong University, 209, Neungdong-ro, Gwangjin-Gu, Seoul 05006, Korea; djkim75@sejong.ac.kr

**Keywords:** waste fishing net, fiber pullout behavior, bond strength, fiber-reinforced cementitious composites, bundled fiber

## Abstract

In this study, recycled waste fishing net (WFN) short fibers were proposed to be used as short fiber reinforcements. The pullout resistance of WFN short fibers embedded in cement mortar was investigated by conducting fiber pullout tests. Three types of WFN short fibers and two types of commercial polypropylene (CP) fibers were investigated. To quantitatively compare the pullout resistance of WFN short fibers and CP fibers, pullout parameters, including peak pullout load (peak bond strength), peak fiber stress, slip at peak load, and pullout energy (equivalent bond strength) of the pullout specimens, were analyzed. In addition, the analysis of fiber images, captured by using a stereoscopic digital microscope, before and after pullout tests, elucidated the different mechanisms of fiber pullout corresponding to the type of fibers. The bundled structures of the WFN fibers generated mechanical interaction between fiber and matrix during fiber pullout; consequently, they produced higher bond resistance and more damage on the surface of fibers after the pullout. Therefore, the bundled WFN fibers showed comparable pullout resistance with CP fibers.

## 1. Introduction

Fiber-reinforced cement composites (FRCCs) have been developed since the 1970s to prevent the brittle failure of cement composites [1,2], and various types of reinforcing fibers have been developed to enhance the tensile strength and toughness of cement composites [3]. Natural, synthetic, and steel fibers have been used [4,5,6,7,8,9], and their bond mechanism has been found to be strongly dependent upon the material and geometry of short fibers. Synthetic fibers generally have lower tensile strength and modulus of elasticity than steel fibers; however, they have excellent corrosion resistance [10]. FRCCs are still more expensive than normal concrete; thus, the use of FRCCs is limited to specific structures, such as high-rise buildings, protection facilities, tunnel lining, and structural foundations [11,12]. To reduce the cost of FRCCs, many researchers have tried to use recycled fibers as short fiber reinforcements in the cementitious matrix [13,14,15,16,17,18]. However, the recycling process requires non-negligible cost, and the performance of recycled fibers is not comparable to that of commercial synthetic fibers [18].

Thus, this study proposed to use waste fishing net (WFN) short fibers as short fiber reinforcements instead of commercial fibers because the use of WFN would be beneficial for reducing the costs and for preventing environmental pollution. Several researchers have previously investigated whether the short fibers produced from WFN, one of the main marine debris, could replace commercial synthetic macro-fibers [19,20,21,22]. Bertelsen and Ottosen [19] compared the performance of WFN fibers with commercial polypropylene (CP) macro-fibers to enhance the tensile resistance of cement mortar. They reported that there was no significant difference in the tensile strengths of WFN and CP fibers, but the stiffness of WFN fiber was lower than that of CP fiber. Spadea et al. [20] reported that FRCCs using nylon-WFN fiber exhibited ductile behavior when subjected to tensile loads and concluded that WFN fiber could be used as tensile reinforcement. Orasutthikul et al. [21] investigated the effectiveness of both straight and knotted WFN fibers as the tensile reinforcement in a fiber-reinforced mortar. They reported that the use of knotted WFN fibers led to relatively higher frictional resistance between the fibers and cement matrix than straight WFN fiber, which showed low frictional resistance. In contrast, those fibers had some disadvantages, such as very pure fiber distribution and low workability [21].

Although previous research has revealed that WFN fibers could enhance the tensile strength of cementitious composites, the interfacial bond strength between WFN fibers and the cement-based matrix is not fully understood yet. Thus, this study focused on further understanding the interfacial bond strength between WFN fibers and mortar by conducting single fiber pullout tests. The specific objectives were to (1) investigate the pullout resistance of the WFN and CP fibers embedded in cement mortar, (2) discover the effect of fiber geometry on the bond strength, and (3) compare the reinforcement effectiveness of WFN fibers with that of CP fibers.

## 2. Background

Synthetic fibers can be generally classified into micro- and macro-fibers corresponding to the size of fibers. The diameter of micro-fibers is between 5 and 100 µm, and the length is between 5 and 30 mm [23], whereas the cross-sectional area of macro-fibers is normally 0.6 to 1 mm^2^, and the length is between 30 and 60 mm [24]. Micro-fibers are generally added to cement mortar to enhance the tensile strength, whereas macro-fibers are effective for the ductility of cementitious composites [25].

**Figure 1:**
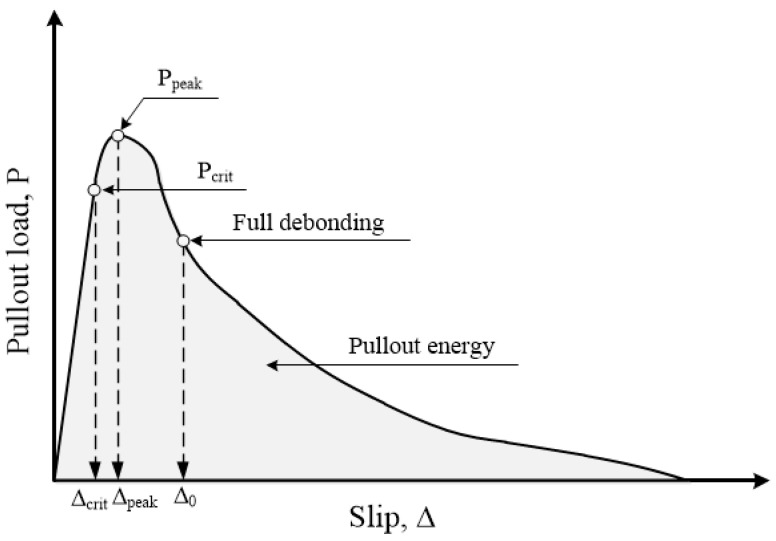
Typical single-fiber pullout behavior [26].

Figure 1 shows the pullout behavior of straight fibers embedded in cement mortar. The interfacial bond between fiber and cement mortar governs the pullout resistance of the embedded fibers. The components of the interfacial bond include chemical, frictional, and mechanical bonds [10]. A chemical bond occurs when fibers adhere during the hydration process, while a frictional bond would be generated due to the surface roughness between the fibers and cement mortar. A mechanical bond occurs when the fiber geometry is deformed during the process of fiber pullout.

**Figure 2:**
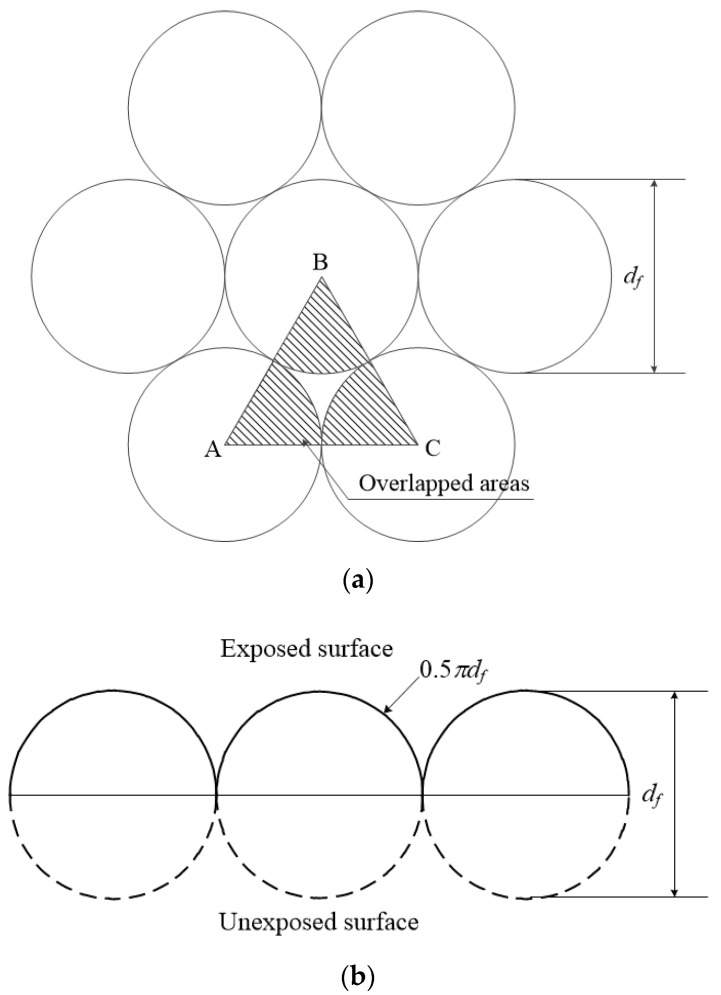
Exposed surface of a multifilament bundled fiber [27]: (**a**) Unit triangular element in a hexagonally packed bundle; (**b**) Surface roughness of a bundled fiber.

Li et al. [27] studied the effects of the inclination angle, bundling, and surface treatment on synthetic fiber pullout from a cement matrix and suggested that fiber bundling should be minimized for efficacy. They suggested a theoretical analysis of a bundled fiber. The ratio of the fiber area, *r_A_*, in the bundle can be calculated by Equation (1), as shown in Figure 2a.

(1) rA=Overlapped areasArea ABC=12·π4·df212df2sin60°=0.907

where the overlapped areas are the areas of the fiber cross-sections inside the element ABC shown in Figure 2.

The cross-sectional area of the bundle can be expressed by Equation (2).

(2) $$\pi R^{2}=\frac{0.25\pi d_{f}{}^{2}N}{r_{A}}$$

where *N* is the total number of fibers, and *R* is the estimated radius of the bundled fiber, which can be obtained from:

(3) $$R=\sqrt{\frac{N}{r_{A}}}\frac{d_{f}}{2}$$

For a bundled fiber, the total perimeter (*p_total_*) is represented by Equation (4).

(4) $$P_{total}=\pi d_{f}N$$

Because the exposed surface of the bundled fiber is composed of a semicircle, as shown in Figure 2b, the ratio of the circumference of a semicircle (0.5*πd_f_*) to its diameter (*d_f_*) represents the bundle surface roughness. Therefore, the exposed perimeter of a bundled fiber (*p_exposed_*) is given by Equation (5).

(5) $$P_{exposed}=2\pi R\frac{0.5\pi d_{f}}{d_{f}}=\pi^{2}R$$

Ferrara et al. [28] conducted studies of high-resolution SEM images as part of morphological identification, which were used to compare the cross-section and perimeter of both dry and impregnated bundled fibers. They concluded that impregnation significantly reduced the perimeter of bundled fibers. Singh et al. [10] investigated the pullout behavior of polypropylene (PP) fibers from a cementitious matrix and concluded that the smooth surface of the PP fibers resulted in a lack of friction and, consequently, a weak bond. Aljewifi et al. [29] reported that the pullout behavior of a glass multifilament fiber embedded in the mortar was determined by the yarn structure, sizing, embedment length, and condition. Di Maida et al. [30] investigated synthetic PP macro-fibers treated with nano-silica to improve the bond characteristics despite their poor interfacial bond strength; however, this method is costly. Recently, Park et al. [22] investigated the mechanical behavior of bundled WFN FRCCs subjected to direct tensile loading and concluded that the tensile strength of the WFN fibers could be comparable to that of CP FRCCs. Therefore, this study investigated the bond strength between bundled WFN fibers and mortar to identify the tensile strength of bundled WFN FRCCs in the previous paper [22].

## 3. Materials Preparation

**Figure 3:**
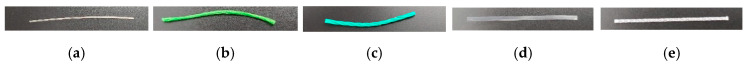
Geometry of the WFN fibers and CP fibers: (**a**) WFN1; (**b**) WFN2; (**c**) WFN3; (**d**) CP1; (**e**) CP2. WFN, waste fishing net; CP, commercial polypropylene.

Table 1 provides the composition of high-strength mortar. The compressive and tensile strength of the high-strength mortar was measured at 28 d. The compressive strength of the mortar was measured according to ASTM C109 [31], while the tensile strength was obtained by using the bell-shaped tensile specimens and following the same method and procedure used by Park et al. [32]. Table 2 summarizes the properties of the WFN and CP short fibers. The WFNs were collected from the seabed near Dalpo Port, Ulsan, South Korea. After removing the contaminants from the collected WFNs, the WFNs were cut into 40 mm short fibers. The detailed procedure for making WFN short fibers is provided by Park et al. [22]. WFN1 was a straight monofilament fiber, as can be seen in Figure 3a, while WFN2 and WFN3 were the bundled multifilament fibers shown in Figure 3b,c. The difference between WFN2 and WFN3 was the number of filaments, which was 18 and 30 for WFN2 and WFN3, respectively. The CP1 fibers (Strux 90/40, GCP Applied Technologies Inc., Cambridge, MA, USA) had surficial scratches, while the CP2 fibers (BT50, GCP Applied Technologies Inc., Cambridge, MA, USA) was embossed, as shown in Figure 3d,e.

A Hobart mixer with 20 L capacity was used to prepare the mortar. Cement and silica sand were dry-mixed for 2 min. Water and superplasticizer, which is the polycarboxylate ether containing 25% solid content, were then gradually added for 1 min and further mixed for 2 min. Before casting, fibers were fixed in a mold to provide 10 mm embedment length, and the specimens were vibrated slightly during the casting. After casting, the specimens were covered with plastic sheeting and stored at room temperature for 24 h before demolding. After demolding, the specimens were water cured at 20 ± 2 °C for 28 d. All specimens were tested under dry conditions at 28 d.

## 4. Test Setup and Procedure

**Figure 4:**
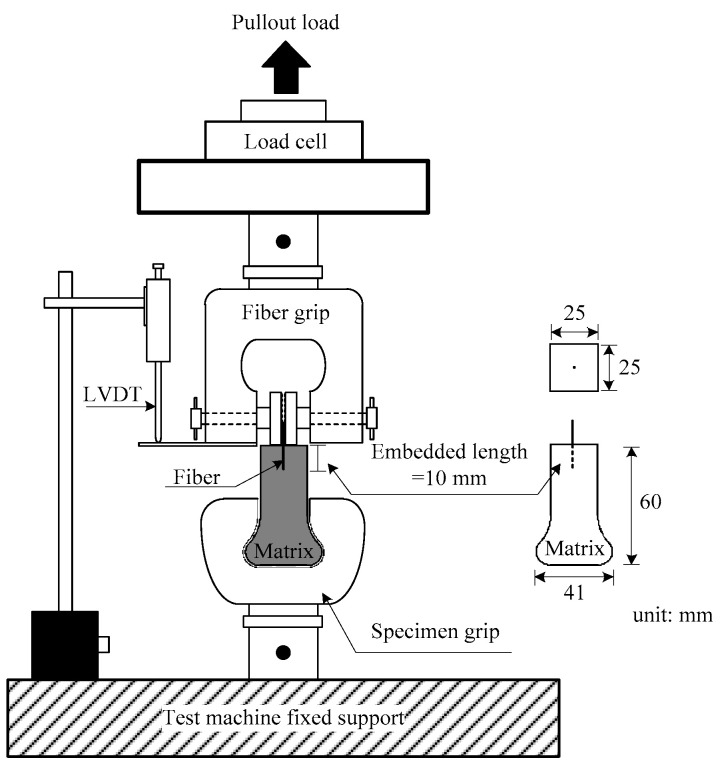
Single-fiber pullout test setup [26].

A universal testing machine with a capacity of 5 kN was used for the single-fiber pullout tests, which were performed at a displacement rate of 1 mm/min. During the pullout test, the load was measured by the load cell located above the loading frame, while the slip was measured using a linear variable differential transformer (LVDT), as shown in Figure 4. The data frequencies of both the load cell and the LVDT were 5 Hz. The surface of the fibers could be checked by analyzing images taken by stereoscopic microscope (HVM0850; Huvitz, Gyeonggi-do Province, Korea) with a Huvitz Lusis HC30-MU camera (Huvitz, Gyeonggi-do Province, Korea). In this study, a magnification of 2.0× was used with an LED light on the stage plate.

## 5. Results

**Figure 5:**
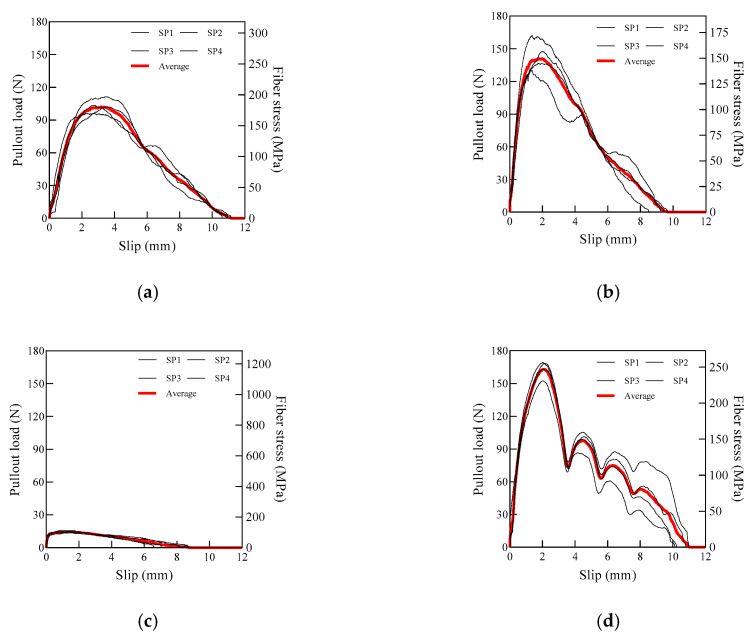
Pullout load versus slip curves of WFN and CP fibers: (**a**) WFN2; (**b**) WFN3; (**c**) CP1; (**d**) CP2.

Figure 5 shows the pullout load versus slip curves of the WFN and CP fibers. The smooth-surfaced WFN1 could not be firmly gripped for the pullout load to be applied; therefore, results were not available (N.A.) for these specimens, and the WFN1 fibers were easily debonded from the mortar. These results agreed with those of Park et al. [22], who reported that WFN fibers with smooth geometry (WFN1 in this study) did not enhance the tensile strength of cement-based materials.

Table 3 shows the peak pullout load (*P_peak_*), fiber stress (*σ_f_*), slip at peak load (Δ_peak_), and pullout energy (*E_P_*) values obtained for the samples. The values of *P_peak_*, Δ_peak_, and *E_P_* were determined from the load versus slip curves, as can be seen in Figure 1, while *σ_f_* was calculated using Equation (6).

(6) $$\sigma_{f}=P_{peak}/A_{f}$$

where *A_f_* is the cross-sectional area of the fiber.

**Figure 6:**
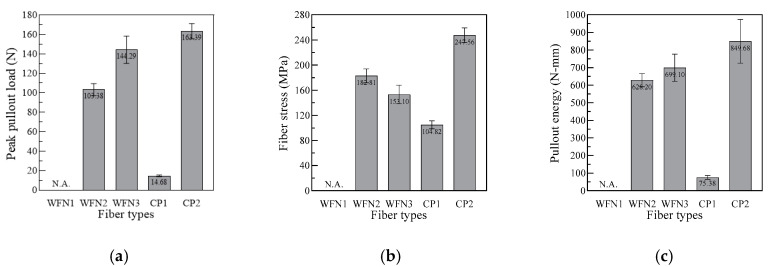
Effect of using different fiber types on the pullout resistance: (**a**) Peak pullout load; (**b**) Fiber stress; (**c**) Pullout energy.

CP2 produced the highest *P_peak_* value of 163.39 N, as can be seen in Figure 6a, whereas CP1 showed the lowest *P_peak_* value of 14.68 N. The difference in *P_peak_* of the CP fibers was caused by the geometry of the synthetic fibers. The embossed fibers, such as CP2, had surface deformation on the fibers, which increased the friction between the fiber and the matrix [33]. However, the smooth fibers, such as CP1, did not. Among the WFN fibers, WFN3 fibers generated the highest *P_peak_* value (144.29 N), and WFN2 produced a *P_peak_* value of 103.38 N. However, the *P_peak_* value was not appropriate for comparing the pullout resistance because the fibers investigated had different cross-sectional areas. Therefore, *σ_f_*, the peak fiber stress during the fiber pullout, was calculated, as provided in Table 3. The *σ_f_* of the WFN2, WFN3, CP1, and CP2 fibers was 182.81, 153.09, 104.82, and 247.56 MPa, respectively; the CP2 fibers obtained the highest σ_f_ due to their high tensile strength of 450 MPa. Further, the WFN2 and WFN3 fibers obtained higher σ_f_ values than that of the CP1 fibers, as shown in Figure 6b; however, the tensile strength values of the WFN fibers were lower than those of the CP fibers. The peak fiber stress was related to the interfacial bond between the fiber and matrix. The σ_f_ values of WFN2 and WFN3, shown in Table 3, reached the tensile strength, as shown in Table 2, while the σ_f_ values of CP1 and CP2 represented only 17% and 55% of their tensile strengths, respectively.

As shown in Figure 6c, the total energy required for fiber pullout from the matrix, *E_P_*, of WFN2, WFN3, CP1, and CP2 was 628.19, 699.09, 75.38, and 849.68 N mm, respectively. Similar to *P_peak_*, *E_P_* was also affected by the fiber geometry. Although there have not been reports on pullout resistance of recycled WFN fibers embedded in cement mortar, the results of previous studies have shown that the tensile strength or flexural strength of the bundled WFN FRCCs is not reduced compared to that of CP FRCCs [22,34]. Therefore, an evaluation of the pure bond strength between the fiber and the mortar, excluding the effect of the fiber geometry, was performed.

## 6. Discussion

The exposed surface area of the fiber bundle that is in contact with the mortar should be calculated when evaluating the bond strength of the WFN fiber multifilament bundles because the exposed surface area is less than the total fiber surface area. Following the method of Li et al. [27], the R and *p_exposed_* of the bundled WFN fibers were calculated, and the perimeter (p) was calculated by the measured total diameter of the fiber, the same as previous study [24]; these values are shown in Table 4. When *p_exposed_* and p were compared, *p_exposed_* always showed a higher value than p because it reflects the surface curvature of the bundled fiber. Therefore, if the bond strength is calculated with p, it may be excessively higher than the actual one. In this study, the bond strength was calculated with *p_exposed_*.

**Figure 7:**
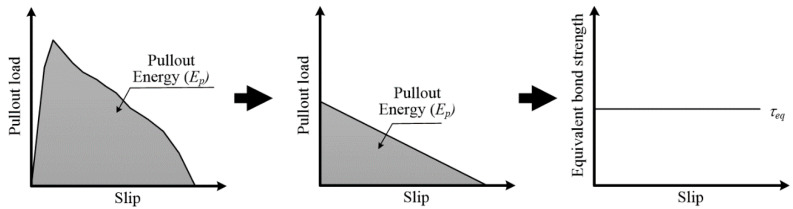
Assumptions underlying the equivalent bond strength calculation [30].

The bond strength between the WFN fiber and mortar could be obtained using the parameters of fiber geometry, and the results are provided in Table 5. Two interfacial bond strengths were calculated using Equations (7) and (8) to quantitatively compare the resistance of the WFN and synthetic fibers to pulling out from the mortar. The peak bond strength (*τ_peak_*) was based on *P_peak_* and was the maximum bond strength during fiber pullout. The equivalent bond strength (*τ_eq_*) was based on the *E_p_* and was derived assuming the bond strength remains constant over the entire embedment length, as shown in Figure 7 [35].

(7) $$\tau_{peak}=\frac{P_{peak}}{p_{exposed}L_{em}}$$

(8) $$\tau_{eq}=\frac{2E_{p}}{p_{exposed}L_{em}{}^{2}}$$

where *L_em_* is the embedded length of the fibers.

**Figure 8:**
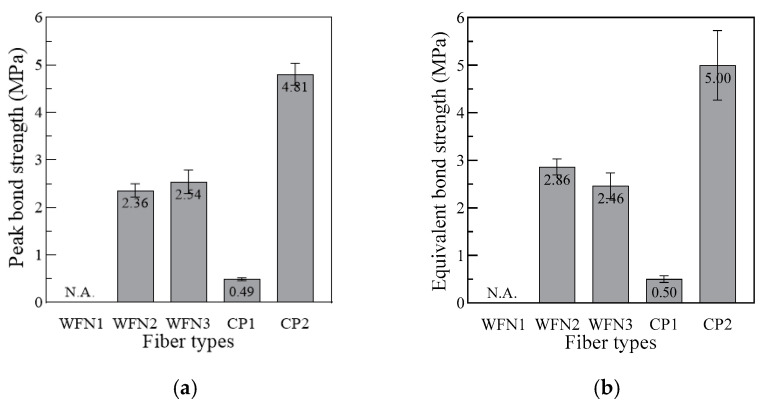
Effect of the different fibers on the: (**a**) Peak bond strength and (**b**) Equivalent bond strength.

Table 5 provides the peak bond strength and equivalent bond strength values, and Figure 8 shows the effect of the different fibers on the bond strength.

**Figure 9:**
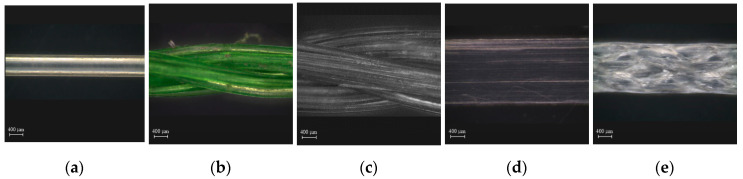
Surfaces of the macro-fibers before the pullout testing: (**a**) WFN1; (**b**) WFN2; (**c**) WFN3; (**d**) CP1; (**e**) CP2 [22].

**Figure 10:**
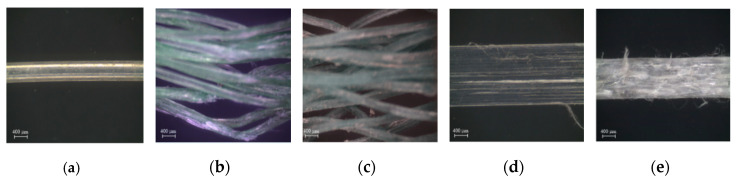
Surfaces of the macro-fibers after the pullout testing: (**a**) WFN1; (**b**) WFN2; (**c**) WFN3; (**d**) CP1; (**e**) CP2.

As shown in Figure 8a, the peak bond strengths of WFN2, WFN3, CP1, and CP2 were 2.36, 2.54, 0.49, and 4.81 MPa, respectively. CP2 had the highest peak bond strength, while CP1 had the lowest. The peak bond strength values of both WFN2 and WFN3 were higher than those of CP1. The fiber surface images shown in Figure 9 and Figure 10 were obtained using a microscope. Figure 9 shows the geometry and surface of each fiber prior to the pullout test [22]. Until the critical load (P_crit_ in Figure 1), the main bond mechanism was the chemical bonding caused by mortar hydration. The interface between the fiber and mortar was separated at the outside of the specimen as *P_peak_* was attained. Figure 10 provides images of the fiber surfaces after pullout testing, which showed that the surface of all the fibers except WFN1, for which a pullout load could not be obtained, were damaged during fiber pullout. The fiber damage resulting from the separation of the fiber and mortar determined the peak bond strength. As shown in Figure 10d, CP1 experienced the minimum amount of surface damage among the samples tested.

**Figure 11:**
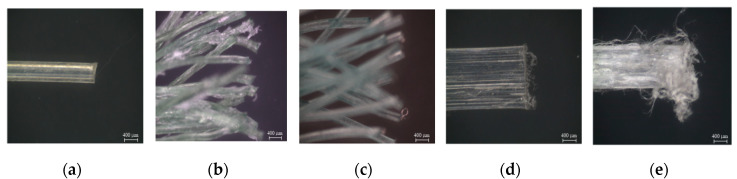
Ends of the macro-fibers after the pullout testing: (**a**) WFN1; (**b**) WFN2; (**c**) WFN3; (**d**) CP1; (**e**) CP2.

As shown in Figure 8b, the equivalent bond strengths of WFN2, WFN3, CP1, and CP2 were 2.86, 2.46, 0.50, and 5.00 MPa, respectively. Similar to the peak bond strength, CP2 had the highest equivalent strength, while CP1 had the lowest. Unlike the peak bond strength, the equivalent bond strength of WFN2 was higher than that of WFN3. The condition of the fiber end after debonding reflected these results. Figure 11 shows the ends of the fibers after the pullout test. The end of the WFN2 fiber showed surface damage, while the WFN3 fiber did not, indicating that the WFN2 fiber experienced higher stress as the tensile strength of the fiber was reached. Further, the WFN2 fiber slipped more than the 10 mm embedment length, as shown in Figure 5a. Therefore, the equivalent strength of WFN2 was higher than that of WFN3.

## 7. Conclusions

This study investigated the interfacial bond strength of five types of synthetic macro-fibers: monofilament WFN fiber (WFN1), two types of multifilament bundled WFN fiber (WFN2 and WFN3), and two types of CP fibers (CP1 and CP2) embedded in cement mortar. The peak bond strength based on the pullout load and the equivalent bond strength based on the pullout energy were calculated to compare the bond characteristics without the influence of the fiber geometry. The following conclusions can be drawn:

CP2 demonstrated the best pullout resistance, in terms of both peak pullout load and pullout energy, because of its high tensile strength (450 MPa) and the extensive fiber damage present after fiber pullout. Although CP1 had the highest tensile strength (620 MPa), it obtained the lowest pullout resistance values because there was almost no damage to the fiber after fiber pullout.

WFN1 could not be tested because its smooth surface prevented it from generating a mechanical bond. Therefore, monofilament WFN would not be recommended for cement mortar reinforcement.

The bundled WFN fiber had a greater contact area with the mortar than did WFN1 and generated a mechanical bond that caused significant damage to both WFN2 and WFN3 during the fiber pullout. Therefore, WFN2 and WFN3 demonstrated higher bond strengths than CP1, which had a smooth surface, and, therefore, less damage to fiber after the pullout.

Among the bundled WFN fibers tested, WFN2 was more efficient than WFN3 because the fiber stress and equivalent bond strength of WFN2 were higher than those of WFN3. Consequently, bundled WFN could be used to reinforce cement mortar.

This study found that due to their high bond strength, bundled WFN fibers could mechanically replace CP fibers for preventing the brittle destruction of cement mortar. However, before utilizing WFN in the construction industry, evaluations of the effect of using the WFN fiber on the durability of cement-based materials are required.

## Figures and Tables

**Table 1:** Matrix composition by weight ratio and compressive strength [22].

Cement (Type I)	Water	Silica Sand	Superplasticizer	Compressive Strength (MPa)	Tensile Strength (MPa)
1.0	0.45	1.5	0.0009	71.9	1.53

**Table 2:** Properties of the synthetic fibers.

Notation	Fiber Type	Diameter of Filament (mm)	Number of Filaments	Embedded Length (mm)	Tensile Strength (MPa)
WFN1	Monofilament	0.45	1	10	305
WFN2	Multifilament (Bundled)	0.2	18	10	188
WFN3	Multifilament (Bundled)	0.2	30	10	173
CP1	Smooth	1.4 × 0.1 ^a^	1	10	620
CP2	Embossed	1.1 × 0.6 ^a^	1	10	450

WFN = Waste Fishing Net; CP = Commercial Polypropylene; ^a^ Rectangular section.

**Table 3:** Pullout test results.

Notation	Specimens	Peak Pullout Load *P_peak_* (N)	Fiber Stress *σ_F_* (MPa)	Slip at Peak Load Δ_Peak_ (mm)	Pullout Energy *E_P_* (N·mm)
WFN1	SP1	N.A.	N.A.	N.A.	N.A.
	SP2	N.A.	N.A.	N.A.	N.A.
	SP3	N.A.	N.A.	N.A.	N.A.
	**Average**	**N.A.**	**N.A.**	**N.A.**	**N.A.**
WFN2	SP1	102.10	180.55	3.63	623.64
	SP2	111.45	197.09	3.55	664.57
	SP3	96.30	170.30	2.69	646.89
	SP4	103.65	183.29	2.77	577.68
	**Average**	**103.38**	**182.81**	**3.16**	**628.19**
WFN3	SP1	147.55	156.56	1.96	693.67
	SP2	162.45	172.36	1.38	809.92
	SP3	136.95	145.31	1.74	658.35
	SP4	130.20	138.15	1.36	634.44
	**Average**	**144.29**	**153.09**	**1.61**	**699.09**
CP1	SP1	14.50	103.57	1.34	66.01
	SP2	15.70	112.14	1.19	83.87
	SP3	13.50	96.43	1.17	65.50
	SP4	15.00	107.14	0.87	86.15
	**Average**	**14.68**	**104.82**	**1.14**	**75.38**
CP2	SP1	168.65	255.53	2.14	888.23
	SP2	163.20	247.27	2.07	818.15
	SP3	169.25	256.44	2.00	994.46
	SP4	152.45	230.98	2.06	697.86
	**Average**	**163.39**	**247.56**	**2.07**	**849.68**

N.A.: Not available.

**Table 4:** Theoretical consideration of multifilament WFN fibers.

Notation	*d_f_* (mm)	*N* (ea)	Total Diameter (mm)	*r_A_* -	*R* (mm)	*p_exposed_* (mm)	*p* (mm)
WFN2	0.2	18	1.0	0.907	0.445	4.397	3.142
WFN3	0.2	30	1.5	0.907	0.575	5.676	4.712

**Table 5:** Calculated bond strength.

Notation	Specimens	Peak Bond Strength (MPa)	Equivalent Bond Strength (MPa)
WFN2	SP1	2.32	2.84
	SP2	2.53	3.02
	SP3	2.19	2.94
	SP4	2.36	2.63
	**Average**	**2.36**	**2.86**
WFN3	SP1	2.60	2.44
	SP2	2.86	2.85
	SP3	2.41	2.32
	SP4	2.29	2.24
	**Average**	**2.54**	**2.46**
CP1	SP1	0.48	0.44
	SP2	0.52	0.56
	SP3	0.45	0.44
	SP4	0.50	0.57
	**Average**	**0.49**	**0.50**
CP2	SP1	4.96	5.22
	SP2	4.80	4.81
	SP3	4.98	5.85
	SP4	4.48	4.11
	**Average**	**4.81**	**5.00**
